# Association of CDKAL1 rs7754840 with gestational diabetes mellitus: a systematic review and meta-analysis

**DOI:** 10.3389/fendo.2026.1947796

**Published:** 2026-09-10

**Authors:** Ke Yi, Ao Wang, Dan Shan

**Affiliations:** 1 Department of Gynaecology and Obstetrics, Hubei Provincial Key Laboratory of Occurrence and Intervention of Rheumatic Diseases, Minda Hospital of Hubei Minzu University, Hubei Minzu University, Enshi, Hubei, China; 2 Key Laboratory of Birth Defects and Related Diseases of Women and Children (Sichuan University),Ministry of Education,West China Second University Hospital, Sichuan University, Chengdu, Sichuan, China

**Keywords:** CDKAL1, GDM, meta-analysis, polymorphisms, rs7754840

## Abstract

**Background and purpose:**

Gestational diabetes mellitus (GDM) is a widespread condition during pregnancy with substantial fetal and maternal morbidity. However, the correlation between GDM risk and the CDKAL1 rs7754840 polymorphism is inconsistent.This study aimed to comprehensively evaluate this association through an updated systematic review and meta-analysis.

**Methods:**

This systematic review and meta-analysis was conducted in accordance with the PRISMA guidelines. A comprehensive literature search of case-control studies was conducted in CNKI, Embase, PubMed, Scopus, and Web of Science from database inception to December 17, 2025. Five genetic models were selected, along with their odds ratios (ORs) and 95% confidence intervals. Subsequently, stratified analyses were performed by ethnicity and according to diagnostic criteria for GDM. Heterogeneity, publication bias, and trial sequential analysis (TSA) were assessed.

**Results:**

The meta-analysis included 18 studies comprising 8,025 women with GDM and 8,605 controls. Pooled analyses showed an association between the CDKAL1 rs7754840 polymorphism and increased GDM risk, with moderate to substantial between-study heterogeneity across genetic models. Stratified analyses showed consistent associations in Asian populations (15 studies; 7,012 cases/7,921 controls), whereas evidence in Caucasian populations (3 studies; 1,013 cases/684 controls) was inconclusive. Heterogeneity varied across GDM diagnostic criteria, with lower heterogeneity observed among studies using the IADPSG (2010) criteria. TSA showed that the cumulative Z-curve crossed the monitoring boundary but did not reach the required information size, indicating supportive but inconclusive evidence.

**Conclusions:**

This meta-analysis supports the association between GDM risk and CDKAL1 rs7754840 in Asian populations.

**Systematic Review Registration:**

https://www.crd.york.ac.uk/PROSPERO/view/CRD420251270398, identifier CRD420251270398.

## Introduction

Glucose intolerance, first diagnosed in pregnancy, commonly known as gestational diabetes mellitus (GDM), is increasingly prevalent all over the world (1, 2). Associations between GDM and adverse pregnancy outcomes, including cesarean delivery, macrosomia, and preeclampsia, as well as enduring health risks to mothers and their children, such as type 2 diabetes mellitus and a child’s future development of metabolic disorders (3–5). These short- and long-term consequences highlight GDM as an important and growing public health concern and underscore the need to elucidate its underlying determinants (6).

Pregnancy is characterized by progressive physiological insulin resistance, and inadequate pancreatic β-cell compensation for this increased insulin demand leads to the development of GDM (7, 8). Increasing evidence indicates that this failure of metabolic adaptation is partly determined by genetic susceptibility, particularly variants affecting pancreatic β-cell function and insulin secretion (9, 10). Among the genetic loci implicated in glucose metabolism, CDKAL1 has emerged as a biologically plausible candidate gene for GDM susceptibility (11). CDKAL1 encodes a methylthiotransferase involved in post-transcriptional modification of transfer RNA, a process essential for translational fidelity and efficient insulin biosynthesis in pancreatic β cells (12, 13). Variants of the CDKAL1 gene have been previously confirmed in genome-wide association studies (GWAS) for T2DM and have been linked to reduced glucose-stimulated insulin secretion and reduced β-cell function (14, 15). Given the shared pathophysiological features between type 2 diabetes and GDM, these findings suggest that CDKAL1 variants may also contribute to dysglycaemia during pregnancy (16). Among these variants, the rs7754840 polymorphism has attracted particular attention; however, epidemiological studies evaluating its association with GDM risk have yielded inconsistent results across different populations (17–19). The rs7754840 polymorphism is relatively common in the general population, with allele frequencies varying across ethnic groups, and has been widely reported in genome-wide association studies of glucose metabolism and type 2 diabetes.

Associations between polymorphisms in the CDKAL1 gene and the risk of GDM have been explored in previous meta-analyses, particularly rs7754840. Notably, Yu et al. reported supportive evidence for this association; however, their analysis was limited by the relatively small number of eligible studies and a modest overall sample size (20). In addition, the rs7754840-specific analysis was based on a subset of studies reporting genotype data, and substantial between-study heterogeneity remained. Importantly, the influence of different GDM diagnostic criteria on heterogeneity was not systematically evaluated, despite considerable variation in diagnostic standards across studies. Therefore, we conducted an updated systematic review and meta-analysis incorporating newly available evidence and expanding the existing evidence base. A total of 18 eligible case–control studies were included to provide a more comprehensive assessment of the association between the CDKAL1 rs7754840 polymorphism and GDM risk and to further explore potential sources of between-study heterogeneity.

## Materials and methods

### Publication search

This systematic review and meta-analysis was conducted and reported in accordance with the Preferred Reporting Items for Systematic Reviews and Meta-Analyses (PRISMA) guidelines. Given that the included evidence consisted primarily of observational case–control studies, the Meta-analysis of Observational Studies in Epidemiology (MOOSE) recommendations were also considered as a complementary reporting framework.

A comprehensive literature search was conducted in CNKI, Embase, PubMed, Scopus, and Web of Science from database inception to December 17, 2025. A search using free-text terms and controlled vocabulary (e.g., MeSH terms in PubMed and Emtree terms in Embase) was performed for GDM, CDKAL1, and genetic polymorphisms. The search was made using the Boolean operators (AND, OR). **Supplementary Table 1** contains details of the complete search strategies carried out for each database.

This study was registered in the PROSPERO database (CRD420251270398). The eligibility criteria and study selection procedures were specified in the registered protocol before study selection. The review was conducted in accordance with the registered protocol, with any subsequent deviations reported transparently in the manuscript.

### Inclusion and exclusion criteria

Studies were eligible for inclusion if they met the following criteria: (i) they evaluated the association between the CDKAL1 rs7754840 polymorphism and the risk of GDM; and (ii) they provided sufficient data to calculate ORs and 95% CIs. Conference abstracts, conference reports, reviews, meta-analyses, studies unrelated to the CDKAL1 rs7754840 polymorphism, and studies without sufficient data were excluded.

### Data extraction

Data were independently extracted by two reviewers (KY and AW) using predefined criteria. Disagreements were resolved through discussion or consultation with a third reviewer (DS). Full-text of each article was extracted, including: author last name, ethnicity, publication year, country of participants, GDM diagnostic criteria, and sample size, and genotyping method. Ethnicity was defined based on the characteristics of the study participants as reported in the original articles. In cases where ethnicity was not explicitly stated, it was inferred from the study population or geographic location. The diagnostic criteria for GDM applied in the included studies varied according to different international guidelines, and the corresponding definitions and glucose thresholds are summarized in **Supplementary Table 2**. To ensure consistency across studies, allele notation was standardized to a common reference (C/G) for the CDKAL1 rs7754840 polymorphism. When necessary, allele labels reported in the original studies were converted to match this reference to maintain comparability of effect estimates. Study quality was assessed using the Newcastle–Ottawa Scale (NOS), which evaluates selection, comparability, and exposure. The maximum NOS score was 9. Studies with a NOS score ≥6 were considered to be of high quality.

### Statistical analysis

The association between GDM and *the* CDKAL1 rs7754840 polymorphism was investigated using ORs and their 95% CIs. Five genetic models were tested in the study, including 1) the allele model (G as compared to C); 2) the homozygote model (GG vs. CC); 3) the heterozygote model (GC vs. CC); 4) the dominant model (GG + GC vs. CC); and 5) the recessive model (GG vs. GC + CC). Subgroup analyses were prespecified and conducted by ethnicity and by diagnostic criteria for GDM. These factors were selected *a priori* because of their potential influence on effect estimates and between-study heterogeneity.

Between-study heterogeneity was assessed using Cochran’s Q test and the I² statistic, with I² values of approximately 25%, 50%, and 75% indicating low, moderate, and high heterogeneity, respectively (21). Random-effects models were used when substantial heterogeneity was present (I² ≥ 50%) (22). Hardy–Weinberg equilibrium (HWE) in the control group of each included study was assessed using a chi-square test. Deviation from HWE was defined as P < 0.05. HWE status was assessed as a study-level methodological characteristic. All studies meeting the eligibility criteria were included in the primary analysis, and sensitivity analyses excluding studies with deviation from HWE were performed to assess the robustness of the pooled estimates. Leave-one-out sensitivity analysis was performed by sequentially excluding each study and recalculating the pooled effect estimates to assess the stability and robustness of the meta-analysis results.

To evaluate for publication bias, Begg’s test and Egger’s test were performed, and funnel plots were plotted to visually assess the publication bias. When a minimum of 10 studies were available, these analyses were carried out. A P value < 0.05 was considered indicative of potential publication bias (23, 24). All statistical tests were two-sided and were performed using Stata version 18.0.

### Trial sequential analysis

Trial sequential analysis (TSA) was performed to assess the reliability of the cumulative evidence and to estimate the required information size (RIS). The analysis was conducted with a two-sided type I error rate of 5% and a statistical power of 80%. The cumulative Z-curve was evaluated against the conventional significance boundary and the trial sequential monitoring boundary. TSA software developed by the Copenhagen Trial Unit was used for the analysis.

## Results

### Characteristics of studies

**Figure 1:**
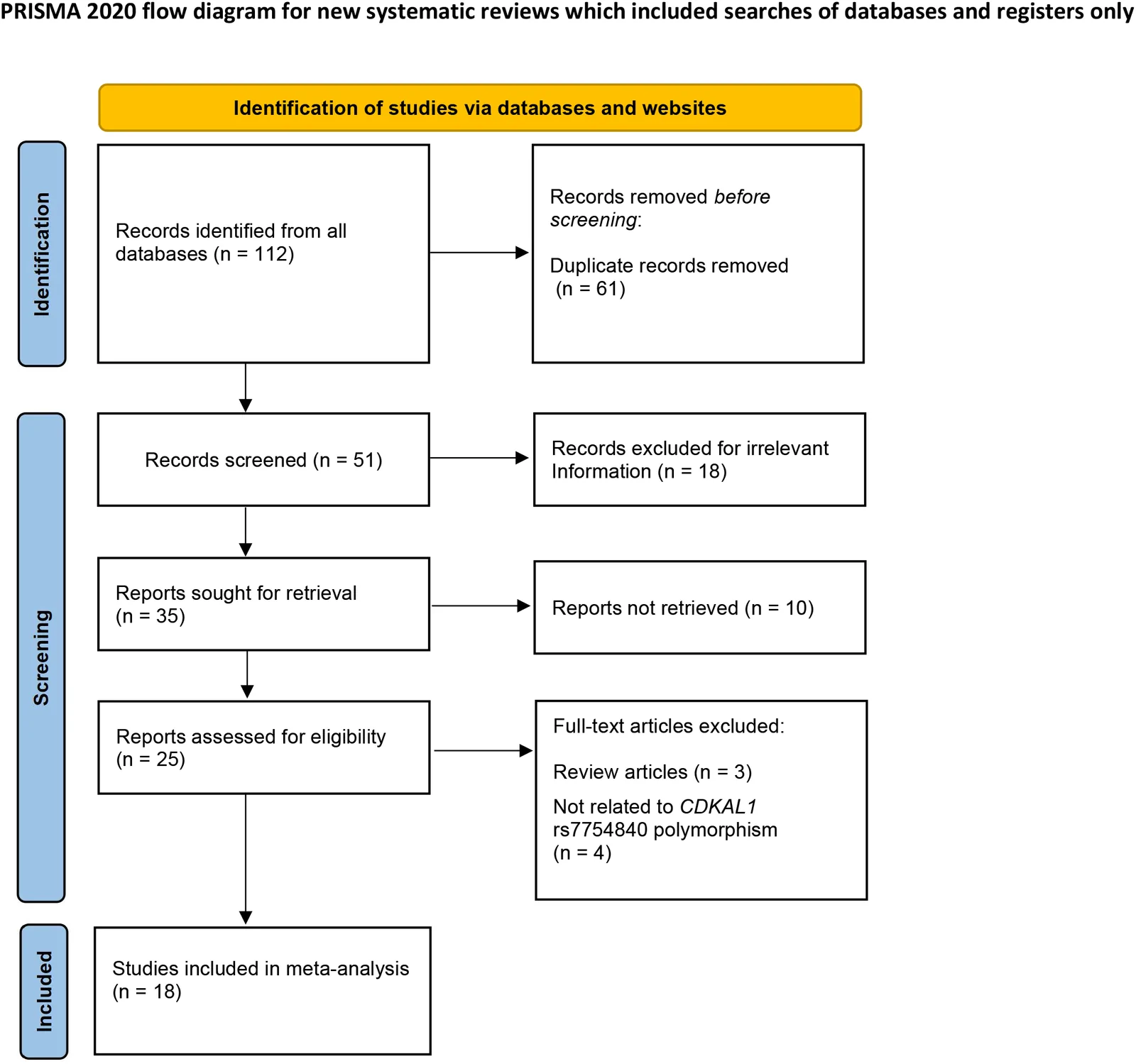
Literature search and study selection procedures used for a meta-analysis of CDKAL1 rs7754840 genetic polymorphism and GDM.

A total of 35 records were initially identified through the literature search. Ten articles were excluded after reviewing titles and abstracts. Full text access to 25 articles was achieved and assessed, and further exclusion of 3 review papers (11, 25, 26), and 4 publication unrelated with the CDKAL1 rs7754840 polymorphism (27–30). Ultimately, 18 case–control studies evaluating the association between the CDKAL1 rs7754840 polymorphism and GDM risk met the predefined eligibility criteria and were included in the meta-analysis (18, 19, 31–46). The study selection process is shown in **Figure 1**.

The main characteristics of the included studies are summarized in **Table 1**. Three studies included Caucasian participants, and 15 included Asian participants. The included studies were conducted in several countries, including China, Russia, India, Bangladesh, Korea, Malaysia, and Egypt.

**Table 1:** Characteristics of studies included in this meta-analysis.

Author	Year	Country	Ethnicity	Sample GDM/control	Diagnostic criteria for GDM	Genotyping methods	NOS score	HWE
Zhang (46)	2009	China	Asian	471/589	ADA(2001)	PCR-RFLP	7	0.872
Cho (19)	2009	Korean	Asian	869/632	ADA(2001)	TaqMan	7	0.653
Aris (40)	2011	Malaysia	Asian	174/114	WHO(1999)	Illumina GoldenGate assay	6	0.072
Wang (37)	2011	China	Asian	725/1039	ADA(2001)	TaqMan	8	0.596
Deng (41)	2013	China	Asian	160/160	ADA(2001)	PCR-RFLP	7	0.592
Hu (43)	2014	China	Asian	176/185	ADA(2001)	SNaPshot	6	<0.001
Kanthimathi (34)	2015	India	Asian	495/910	IADPSG(2010)	MassARRAY	6	0.630
Wu (45)	2015	China	Asian	153/180	IADPSG(2010)	PCR-RFLP	6	0.366
Popova (35)	2017	Russia	Caucasian	278/179	IADPSG(2010)	PCR-RFLP	7	0.141
Li (39)	2017	China	Asian	553/721	IADPSG(2010)	MassARRAY	7	0.545
Noury (18)	2018	Egypt	Caucasian	47/51	ADA(2001)	TaqMan	6	0.746
Xie (38)	2019	China	Asian	964/1021	IADPSG(2010)	MassARRAY	7	0.144
Guo (42)	2019	China	Asian	473/516	IADPSG(2010)	PCR-RFLP	7	0.074
Popova (36)	2021	Russia	Caucasian	688/454	IADPSG(2010)	TaqMan	8	0.313
Amin (31)	2022	Bangladesh	Asian	212/256	IADPSG(2010)	TaqMan	6	0.317
Huang (33)	2023	China	Asian	835/870	IADPSG(2010)	MassARRAY	7	0.661
Wang (44)	2024	China	Asian	252/226	IADPSG(2010)	MassARRAY	7	0.572
Gyan (32)	2025	China	Asian	500/502	IADPSG(2010)	SNPscan	8	0.771

PCR-RFLP, polymerase chain reaction-restriction fragment length polymorphism; GDM, gestational diabetes mellitus; ADA, American Diabetes Association; WHO, world health organization; IADPSG, international association of the diabetes and pregnancy study groups. NOS, Newcastle-Ottawa Scale.

### Quantitative synthesis

**Figure 2:**
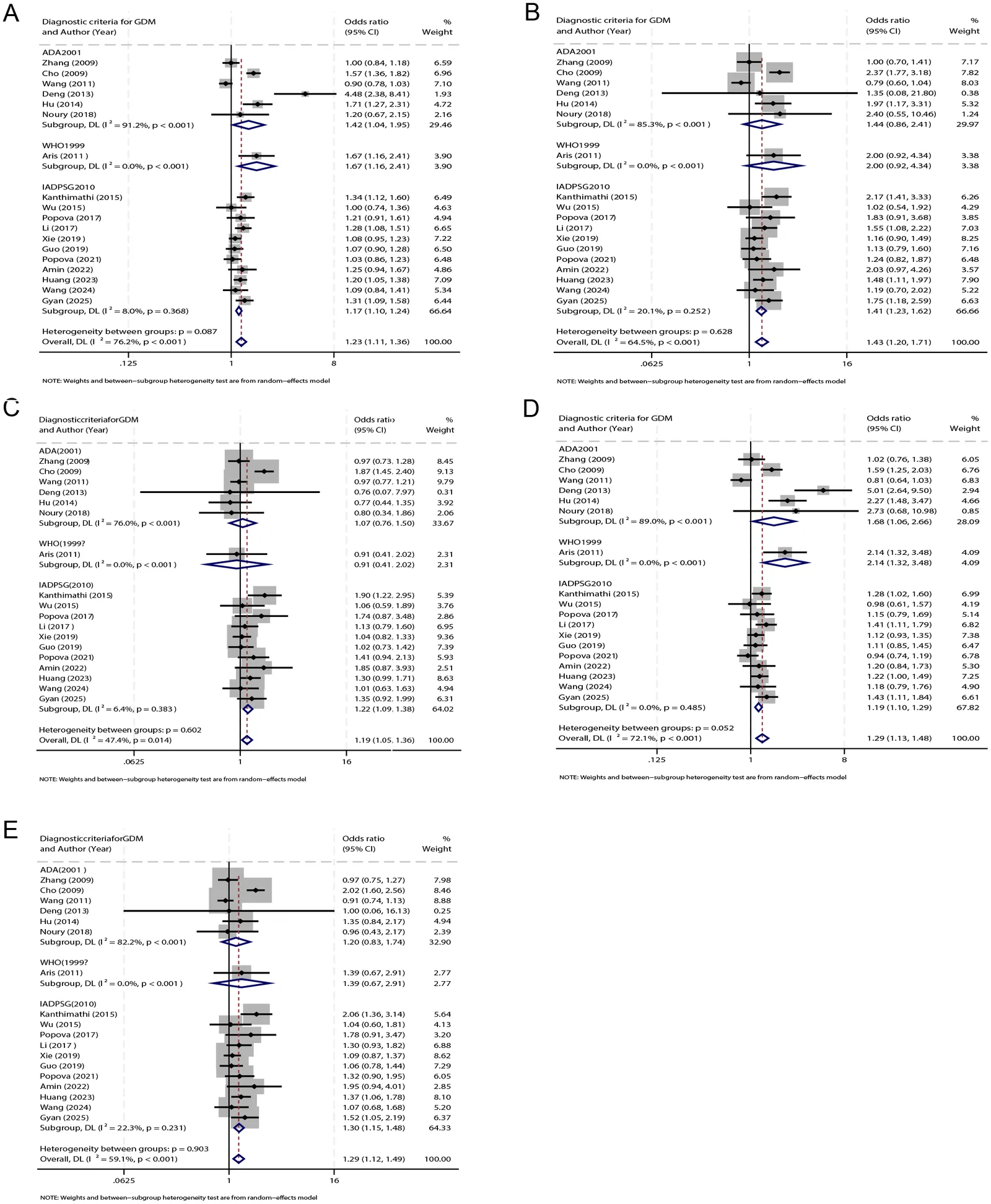
Forest plots of ORs with 95% CIs for CDKAL1 rs7754840 polymorphism and GDM risk using ethnicity subgroup analysis. **(A–E)** show allelic, homozygous, heterozygous, dominant model and recessive models, respectively.

Data from 18 studies comprising 8,025 cases and 8,605 controls were used for quantitative synthesis. The pooled analyses revealed that the CDKAL1 rs7754840 polymorphism was significantly associated with GDM risk across all five genetic models (**Table 2**). The trends were consistent across the Asian population group subjects in the stratified analyses. No significant association was observed among Caucasian individuals, but the data should be interpreted with caution, as only a few studies included this population. Overall, the direction of the pooled effect estimates was generally consistent with an increased risk of GDM across the five genetic models (**Figure 2**).

**Table 2:** Quantitative analyses of the CDKAL1 rs7754840 polymorphism on the GDM risk stratified by enthnicity.

Genetic model			Allele contrast			Homozygote			Heterozygote			Dominant Model			Recessive Model		
Variables	Sample size		G vs. C			GG vs. CC			GC vs. CC			GG+ GC vs. CC			GG vs. GC + CC		
	N^a^	Case/control	OR(95%CI)	P_het_^b^	I²	OR(95%CI)	P_het_^b^	I²	OR(95%CI)	P_het_^b^	I²	OR(95%CI)	P_het_^b^	I²	OR(95%CI)	P_het_^b^	I²
Total	18	8025 / 8605	1.23 (1.11,1.36)	0.001	76.2%	1.43 (1.20,1.71)	0.001	64.5%	1.19 (1.05,1.36)	0.014	47.4%	1.29 (1.13,1.48)	0.001	72.1%	1.29 (1.12,1.49)	0.001	59.1%
Caucasian	3	1013 / 684	1.08 (0.94,1.25)	0.589	0%	1.41 (1.00,1.99)	0.495	0%	1.36 (0.98,1.89)	0.361	1.9%	1.05 (0.80,1.39)	0.249	28.1%	1.35 (0.99,1.84)	0.514	0%
Asian	15	7012 / 7921	1.25 (1.12,1.40)	0.001	79.7%	1.43 (1.17,1.73)	0.001	69.9%	1.18 (1.02,1.36)	0.009	52.6%	1.33 (1.15,1.54)	0.001	74.3%	1.29 (1.10,1.51)	0.001	65.0%

^a^ Number of comparisons.

^b^ P_het_ represents the P value from Cochran’s Q test for heterogeneity.

The NOS scores, as an indicator of the quality of the investigations, are presented in **Supplementary Table 3**.

### Heterogeneity analysis

Significant between-study heterogeneity was observed across all five genetic models (P_heterogeneity_ < 0.05). Accordingly, random-effects models were used for the pooled analyses.

**Figure 3:**
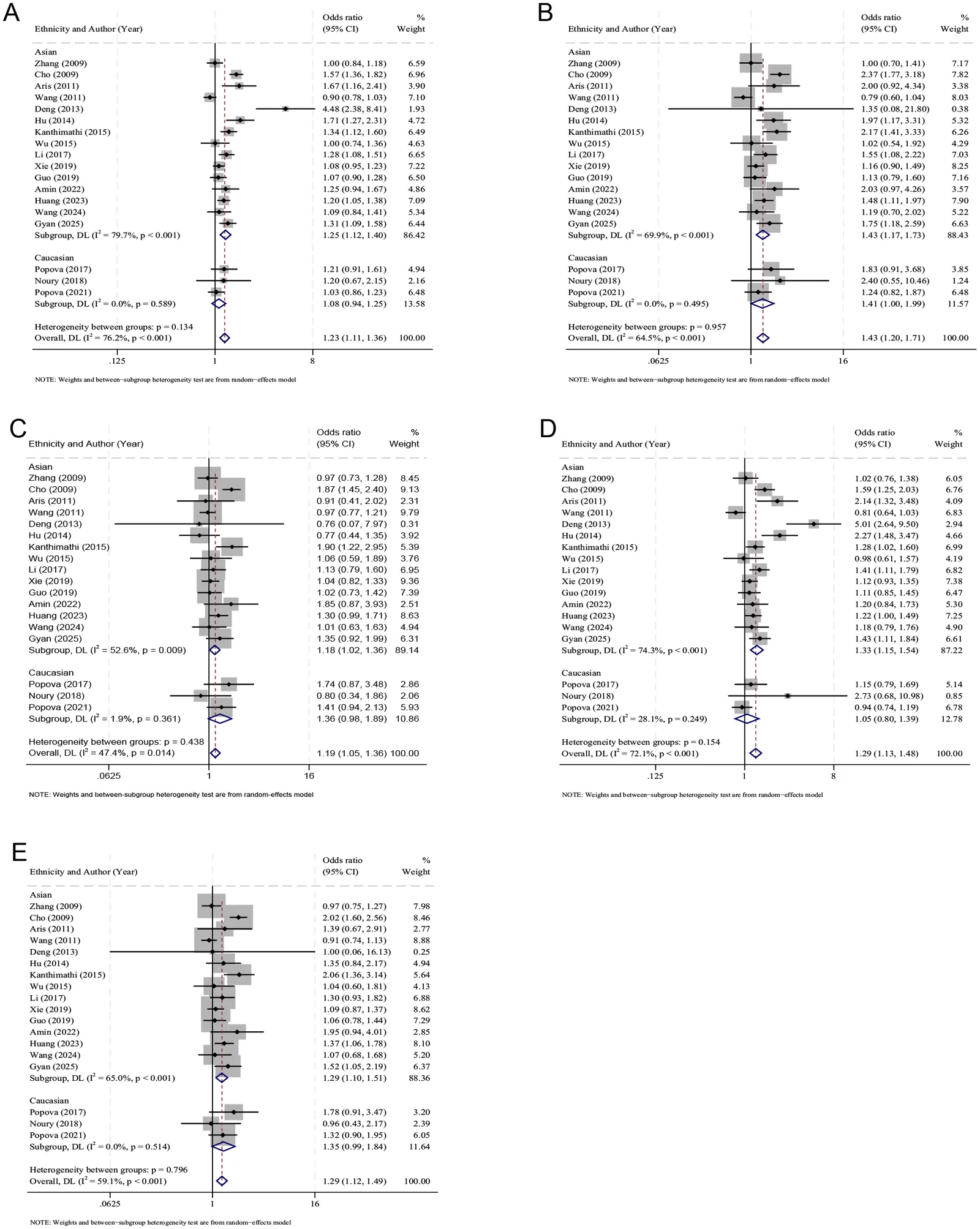
Forest plot for the association between CDKAL1 rs7754840 polymorphism and GDM risk using GDM diagnostic criteria subgroup analysis. **(A–E)** show allelic, homozygous, heterozygous, dominant model and recessive models, respectively.

Subgroup analyses stratified by GDM diagnostic criteria showed substantial heterogeneity among studies using the ADA (2001) criteria, with I² values ranging from 76.0% to 91.2% across the five genetic models (**Table 3**). In contrast, lower heterogeneity was observed among studies using the IADPSG (2010) criteria, with I² values ranging from 0% to 22.3%. Heterogeneity could not be estimated for the WHO (1999) subgroup because only one study was included. These results indicate that the magnitude of between-study heterogeneity varied across diagnostic-criteria subgroups (**Figure 3**).

**Table 3:** Quantitative analyses of CDKAL1 rs7754840 and GDM risk stratified by diagnostic criteria for GDM.

Genetic model			Allele contrast			Homozygote			Heterozygote			Dominant Model			Recessive Model		
Variables	Sample size		G vs. C			GG vs. CC			GC vs. CC			GG+ GC vs. CC			GG vs. GC + CC		
	N^a^	Case/control	OR(95%CI)	P_het_^b^	I²	OR(95%CI)	P_het_^b^	I²	OR(95%CI)	P_het_^b^	I²	OR(95%CI)	P_het_^b^	I²	OR(95%CI)	P_het_^b^	I²
Total	18	8025 / 8605	1.23 (1.11,1.36)	0.001	76.2%	1.43 (1.20,1.71)	0.001	64.5%	1.19 (1.05,1.36)	0.014	47.4%	1.29 (1.13,1.48)	0.001	72.1%	1.29 (1.12,1.49)	0.001	59.1%
ADA(2001)	6	2448/2656	1.42 (1.04,1.95)	0.001	91.2%	1.44 (0.86,2.41)	0.001	85.3%	1.07 (0.76,1.50)	0.001	76.0%	1.68 (1.06,2.66)	0.001	89.0%	1.20 (0.83,1.74)	0.001	82.2%
IADPSG (2010)	11	5403 / 5835	1.17 (1.10,1.24)	0.368	8.0%	1.41 (1.23,1.62)	0.252	20.1%	1.22 (1.09,1.38)	0.383	6.4%	1.19 (1.10,1.29)	0.485	0%	1.30 (1.15,1.48)	0.231	22.3%
WHO (1999)	1	174 / 114	1.67 (1.16,2.41)	NA	NA	2.00 (0.92,4.34)	NA	NA	0.91 (0.41,2.02)	NA	NA	2.14 (1.32,3.48)	NA	NA	1.39 (0.67,2.91)	NA	NA

^a^ Number of comparisons.

^b^ P_het_ represents the P value from Cochran’s Q test for heterogeneity.

GDM, gestational diabetes mellitus; ADA, American Diabetes Association; WHO, world health organization; IADPSG, international association of the diabetes and pregnancy study groups.

### Sensitivity analyses

The pooled effect estimates remained stable in the leave-one-out sensitivity analysis, with no material changes in the magnitude or direction of the associations after sequential exclusion of individual studies (**Supplementary Figure 1**). This indicates that the overall findings were not disproportionately influenced by any single study. One included study (Hu, 2014) deviated from HWE in the control group (43). Sensitivity analysis excluding this study did not materially alter the pooled estimates, indicating that its inclusion had limited influence on the overall findings.

### Publication bias

**Figure 4:**
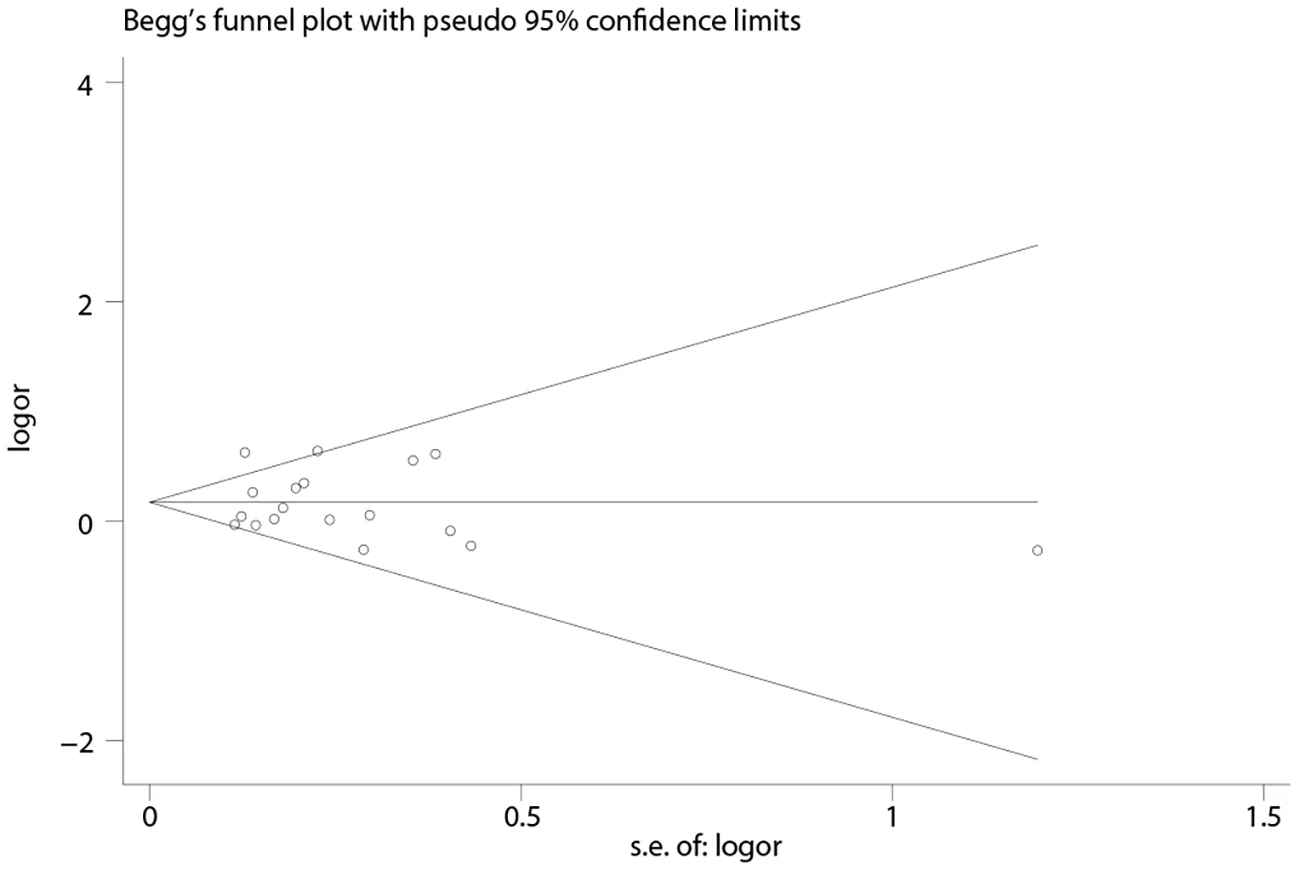
Begg’s funnel plot for publication bias test.

Egger’s test and Begg’s test were used to evaluate the publication bias. A funnel plot (**Figure 4**) was then used to further examine the possibility of publication bias and was found to be roughly symmetric, which further provided no clear evidence of funnel-plot asymmetry. Across the different genetic comparisons, Egger’s and Begg’s tests yielded the following p values: recessive model (Egger’s p = 0.47; Begg’s p = 0.28), heterozygote comparison (Egger’s p = 0.29; Begg’s p = 0.22), allele contrast (Egger’s p = 0.18; Begg’s p = 0.12), dominant model (Begg’s p = 0.11; Egger’s p = 0.46), and homozygote comparison (Egger’s p = 0.98; Begg’s p = 0.44). Overall, these findings suggest no significant evidence of publication bias across the included studies; however, the statistical power of these tests may be limited due to the relatively small number of studies in some comparisons.

### Trial sequential analysis

**Figure 5:**
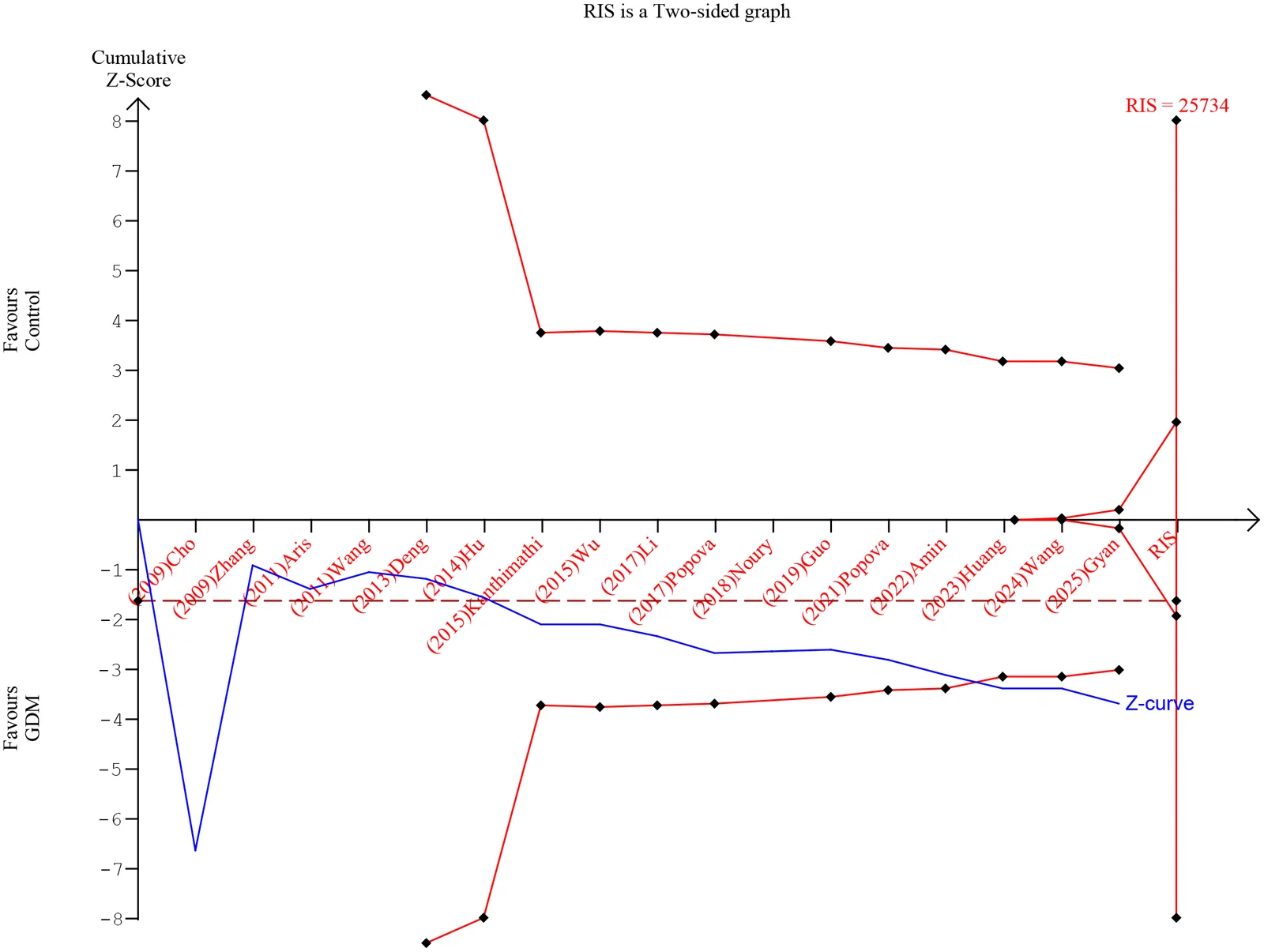
Trial sequential analysis of GDM risk associated with CDKAL1 rs7754840 polymorphism.

To evaluate the quality of the accumulated evidence and minimize the random errors, a trial sequential analysis (TSA) was performed. The cumulative Z-curve crossed both the conventional significance boundary and the trial sequential monitoring boundary (**Figure 5**), although the accrued information size did not reach the required information size. Thus, the TSA supports the observed association, but the evidence should not yet be considered conclusive.

## Discussion

The present meta-analysis included 18 independent case-control studies comprising 8,025 women with GDM and 8,605 controls. The pooled analyses suggested an association between the CDKAL1 rs7754840 polymorphism and GDM risk across several genetic models; however, these findings should be interpreted cautiously because of substantial between-study heterogeneity. Although the observed effect sizes were modest, they are consistent with the polygenic nature of GDM, in which individual genetic variants typically confer small but cumulative effects on disease risk. Therefore, while the CDKAL1 rs7754840 polymorphism alone may have limited clinical utility as a predictive marker, it may contribute to risk stratification when incorporated into multifactorial prediction models alongside clinical and environmental factors.

Stratified analyses according to ethnicity revealed distinct association patterns between the CDKAL1 rs7754840 polymorphism and GDM risk. Across 15 studies involving about 15,000 Asian participants, rs7754840 showed consistent and significant associations with GDM under all genetic models. No statistically significant association was observed with Caucasian populations (but should be interpreted with caution because the number of studies performed in this population was small and the statistical power was correspondingly low). Although the direction of the pooled effect estimates in Caucasian subjects was generally concordant with that observed in Asians, the corresponding confidence intervals were notably wide, suggesting substantial uncertainty. The lack of statistical significance in Caucasian populations may reflect limited statistical power rather than a true absence of genetic effect. These subgroup findings should therefore be interpreted cautiously, particularly for underrepresented populations.

Stratification by GDM diagnostic criteria revealed substantial differences in the magnitude of between-study heterogeneity(**Table 3**). Although the overall pooled analysis demonstrated significant associations across all genetic models, substantial heterogeneity was observed, prompting stratified analyses to explore potential sources.

Studies applying the ADA (2001) criteria showed substantial heterogeneity across the five genetic models (I² = 76.0%–91.2%). In contrast, lower heterogeneity was observed among studies using the IADPSG (2010) criteria (I² = 0%–22.3%). These differences indicate variation in heterogeneity across diagnostic-criteria subgroups but do not establish diagnostic criteria as the independent source of heterogeneity.

The diagnostic differences summarized in **Supplementary Table 2** may provide one possible explanation for the observed variation. The ADA (2001) criteria use a 100-g OGTT and require multiple abnormal values, whereas the IADPSG (2010) criteria use a 75-g OGTT with different glucose thresholds and require only one abnormal value. Such differences may influence case ascertainment and the clinical characteristics of individuals classified as having GDM. However, diagnostic criteria may be correlated with other study-level characteristics, including recruitment period, ethnicity, geographical region, genotyping method, and population characteristics. Therefore, the lower heterogeneity observed in the IADPSG subgroup cannot be attributed solely to the diagnostic criteria. Because the number of included studies was insufficient for reliable multivariable meta-regression, the independent contribution of these factors could not be determined. These subgroup findings should therefore be regarded as exploratory.

This observed association between the CDKAL1 rs7754840 polymorphism and GDM risk is biologically plausible, given established associations of CDKAL1 with glucose metabolism and pancreatic β-cell function. CDKAL1 encodes a methylthiotransferase that is important in the post-transcriptional modification of transfer RNA, which plays a critical role in translational fidelity and insulin biosynthesis (47). Recent functional and translational studies have confirmed that disruption of CDKAL1-mediated tRNA modification leads to impaired proinsulin translation, endoplasmic reticulum stress, and reduced glucose-stimulated insulin secretion in pancreatic β cells (13, 48). Consistent with these mechanistic findings, large-scale genome-wide association studies and post-GWAS analyses continue to identify CDKAL1 variants, including rs7754840, as robust determinants of β-cell dysfunction and impaired insulin secretory capacity (49). While these biological mechanisms support the plausibility of the observed association, the evidence presented in this meta-analysis is observational in nature and does not establish a causal relationship. Further functional and longitudinal studies are required to clarify the underlying mechanisms.

Pregnancy represents a unique physiological state characterized by progressive insulin resistance driven by placental hormones and maternal metabolic adaptations (50). To maintain normoglycaemia, pancreatic β cells must undergo functional compensation during gestation. Genetic variants that subtly impair insulin secretion, such as rs7754840, may therefore exert amplified effects on glycaemic regulation during pregnancy, when β-cell reserve is physiologically challenged (51). This hypothesis is supported by recent population-based studies and updated meta-analyses demonstrating a consistent association between CDKAL1 polymorphisms and GDM across diverse ethnic groups, with modest but reproducible effect sizes (32).

Beyond these established mechanisms, there is additional biological plausibility that the effects of the CDKAL1 rs7754840 polymorphism may differ across individuals and populations. Given that CDKAL1 variants primarily influence insulin secretion rather than insulin resistance, their phenotypic impact is likely to depend on the degree of β-cell stress and functional reserve. Under conditions of increased metabolic demand, such as pregnancy, individuals with limited β-cell compensatory capacity may be more vulnerable to developing dysglycaemia.

Furthermore, differences in genetic background, linkage disequilibrium patterns, and allele frequencies across populations may partly explain the variability in observed associations. It is also possible that gene–environment interactions, including variations in lifestyle, nutritional status, and metabolic risk profiles, modulate the clinical expression of this polymorphism. These factors together provide a biologically plausible framework for understanding both inter-individual variability and ethnic differences in the association between CDKAL1 rs7754840 and GDM risk.

Our study extends the previous meta-analysis by Yu et al. (20). Several studies included in the present meta-analysis overlapped with those analyzed by Yu et al., while additional eligible studies identified after their search period were incorporated. Yu et al. included 10 eligible studies evaluating the CDKAL1 rs7754840 polymorphism, whereas the present meta-analysis included 18 studies, thereby increasing the overall sample size and statistical power. In addition, we further explored potential sources of between-study heterogeneity through stratified analyses according to ethnicity and GDM diagnostic criteria.

Importantly, our study further explored potential sources of heterogeneity through prespecified subgroup analyses. Stratification by GDM diagnostic criteria revealed lower heterogeneity among studies applying the IADPSG (2010) criteria than among those applying the ADA (2001) criteria. However, because diagnostic criteria may be correlated with other study-level characteristics, this finding should be considered exploratory rather than evidence of an independent effect of diagnostic criteria.

Given that the present meta-analysis examined the association between the rs7754840 variant in the CDKAL1 gene and GDM susceptibility, it was not possible to assess the effect of this variant on pregnancy course and/or outcomes due to limited clinical details in the studies included in the analysis. However, the importance of CDKAL1 in β-cell function and insulin secretion suggests that it may also affect the severity of glycaemic dysregulation in pregnancy and, hence, maternal and neonatal outcomes. From a clinical perspective, the CDKAL1 rs7754840 polymorphism may contribute to GDM risk stratification when incorporated into multifactorial prediction models alongside established clinical factors. However, given its modest effect size, its standalone clinical utility is limited, and further validation in prospective studies is required.

Several limitations should be acknowledged. First, most studies adopted a case–control design, which is inherently susceptible to selection bias, despite the generally acceptable methodological quality assessed by the Newcastle–Ottawa Scale. Second, most of the included studies were conducted in Asian populations, with limited representation of other ethnic groups, which may restrict the generalizability of the findings. Third, due to the lack of individual-level data, this meta-analysis could not assess potential gene–environment interactions or the modifying effects of key clinical factors, such as pre-pregnancy body mass index, maternal age, and gestational weight gain, on the association between the CDKAL1 rs7754840 polymorphism and GDM risk.

In addition, detailed clinical information—such as GDM severity, treatment modality, and maternal or neonatal outcomes—was not consistently reported, precluding subgroup analyses based on disease severity or prognosis. Given that these factors may reflect underlying differences in β-cell dysfunction and metabolic burden, it is biologically plausible that CDKAL1 rs7754840 is associated not only with GDM susceptibility but also with more severe glycaemic dysregulation and related complications. Future well-designed studies incorporating detailed clinical phenotyping and outcome data are needed to clarify its potential role in predicting disease severity and adverse pregnancy outcomes.

Furthermore, one included study deviated from HWE in the control group; however, sensitivity analyses excluding this study did not materially alter the results, indicating that its impact on the overall findings was limited. Finally, the limited number of studies precluded reliable multivariable meta-regression. Therefore, the independent contributions of diagnostic criteria and potentially correlated study-level characteristics, including recruitment period, ethnicity, geographical region, genotyping method, and population characteristics, to between-study heterogeneity could not be disentangled. Accordingly, the findings from the subgroup analyses should be considered exploratory.

## Conclusion

This meta-analysis provides supportive evidence for an association between the CDKAL1 rs7754840 polymorphism and GDM risk, particularly in Asian populations; however, these findings should be interpreted with caution due to between-study heterogeneity and methodological limitations. Given the limited number of studies conducted in European populations, further large-scale investigations in diverse ethnic groups, especially among individuals of European ancestry, are warranted to validate the generalizability of these findings and to clarify potential population-specific genetic effects.

## Data Availability

The original contributions presented in the study are included in the article/**Supplementary Material**. Further inquiries can be directed to the corresponding author.
