# Supplementary material for: Association of CDKAL1 rs7754840 with gestational diabetes mellitus: a systematic review and meta-analysis
Source: Front Endocrinol (Lausanne). 2026 Sep 10;17:1947796. doi: 10.3389/fendo.2026.1947796 (PMC13600907; doi:10.3389/fendo.2026.1947796)
Supplement: Supplementary file 1 [file SupplementaryFile1.docx]

**Supplementary Table S1. Search strategies**

**Pubmed**

("Diabetes, Gestational"[Mesh] OR "gestational diabetes mellitus"[Title/Abstract] OR "gestational diabetes"[Title/Abstract] OR GDM[Title/Abstract]) AND ("CDKAL1"[Title/Abstract] OR "CDK5 regulatory subunit associated protein 1-like 1"[Title/Abstract] OR rs7754840[Title/Abstract]) AND ("Polymorphism, Genetic"[Mesh] OR polymorphism*[Title/Abstract] OR SNP*[Title/Abstract] OR "single nucleotide polymorphism*"[Title/Abstract] OR "genetic variant*"[Title/Abstract] OR "gene variant*"[Title/Abstract])

**Embase**

('gestational diabetes mellitus'/exp OR 'gestational diabetes mellitus':ti,ab OR 'gestational diabetes':ti,ab OR gdm:ti,ab) AND (cdkal1:ti,ab OR 'cdk5 regulatory subunit associated protein 1-like 1':ti,ab OR rs7754840:ti,ab) AND ('genetic polymorphism'/exp OR polymorphism*:ti,ab OR snp*:ti,ab OR 'single nucleotide polymorphism*':ti,ab OR 'genetic variant*':ti,ab OR 'gene variant*':ti,ab)

**Web of Science**

TS = (("gestational diabetes mellitus" OR "gestational diabetes" OR GDM) AND (CDKAL1 OR "CDK5 regulatory subunit associated protein 1-like 1" OR rs7754840) AND (polymorphism OR SNP OR "single nucleotide polymorphism" OR "genetic variant" OR "gene variant"))

**Scopus**

TITLE-ABS-KEY (("gestational diabetes mellitus" OR "gestational diabetes" OR GDM) AND (CDKAL1 OR "CDK5 regulatory subunit associated protein 1-like 1" OR rs7754840) AND (polymorphism* OR SNP* OR "single nucleotide polymorphism*" OR "genetic variant*" OR "gene variant*"))

**CNKI (China National Knowledge Infrastructure)**

(主题：(CDKAL1+CDK5调节亚基相关蛋白1+rs7754840)) AND (主题：(variant +polymorphism+基因多态性)) AND (主题：(gestational diabetes mellitus +GDM+妊娠期糖尿病))

Search period: From database inception to December 17, 2025.

**Supplementary Table S2. Diagnostic criteria for GDM across major guidelines**

| **Criteria** | **Glucose load** | **Abnormal values required** | **Fasting threshold (mmol/L)** | **1-h threshold (mmol/L)** | **2-h threshold (mmol/L)** | **3-h threshold (mmol/L)** |
| --- | --- | --- | --- | --- | --- | --- |
| **WHO (1999)** | 75 g OGTT (1-step) | ≥1 abnormal value | ≥7.0 (or prior diabetes) | – | ≥7.8 | – |
| **ADA (2001)** | 100 g OGTT (2-step) | ≥2 abnormal values | ≥5.3 | ≥10.0 | ≥8.6 | ≥7.8 |
| **IADPSG (2010)** | 75 g OGTT (1-step) | ≥1 abnormal value | ≥5.1 | ≥10.0 | ≥8.5 | – |

Abbreviations: OGTT: oral glucose tolerance test; GDM, gestational diabetes mellitus; ADA,American Diabetes Association; WHO,world health organization; IADPSG, international association of the diabetes and pregnancy study groups.

**Supplementary Table S3. Quality assessment of case–control studies included in this meta-analysis^a^.**

| Study | Adequate  definition of  cases | Representativeness  of cases | Selection  of control | Definition of  control | Control for important factor or  additional factor^b^ | Exposure  assessment | Same method of ascertainment  for cases and controls | Nonresponse  rate^c^ | Total  quality  scores^d^ |
| --- | --- | --- | --- | --- | --- | --- | --- | --- | --- |
| Zhang | ★ | ★ | ★ | ★ | ★ | - | ★ | ★ | 7 |
| Cho | ★ | ★ | ★ | ★ | ★ | - | ★ | ★ | 7 |
| Aris | ★ | ★ | - | ★ | ★ | - | ★ | ★ | 6 |
| Wang | ★ | ★ | ★ | ★ | ★★ | - | ★ | ★ | 8 |
| Deng | ★ | ★ | ★ | ★ | ★ | - | ★ | ★ | 7 |
| Hu | ★ | ★ | - | ★ | ★ | - | ★ | ★ | 6 |
| Kanthimathi | ★ | ★ | - | ★ | ★ | - | ★ | ★ | 6 |
| Wu | ★ | ★ | - | ★ | ★ | - | ★ | ★ | 6 |
| Popova | ★ | ★ | ★ | ★ | ★ | - | ★ | ★ | 7 |
| Li | ★ | ★ | ★ | ★ | ★ | - | ★ | ★ | 7 |
| Noury | ★ | ★ | - | ★ | ★ | - | ★ | ★ | 6 |
| Xie | ★ | ★ | ★ | ★ | ★ | - | ★ | ★ | 7 |
| Guo | ★ | ★ | ★ | ★ | ★ | - | ★ | ★ | 7 |
| Popova | ★ | ★ | ★ | ★ | ★★ | - | ★ | ★ | 8 |
| Amin | ★ | ★ | - | ★ | ★ | - | ★ | ★ | 6 |
| Huang | ★ | ★ | ★ | ★ | ★ | - | ★ | ★ | 7 |
| Wang | ★ | ★ | ★ | ★ | ★ | - | ★ | ★ | 7 |
| Gyan | ★ | ★ | ★ | ★ | ★★ | - | ★ | ★ | 8 |

^a^ A study can be awarded a maximum of one star for each numbered item except for the item Control for most important factor or second important factor.

^b^ A maximum of two stars can be awarded for Control for most important factor or second important factor. Studies that controlled for maternal age received one star, whereas studies that controlled for high risk factor (diabetes or pre-pregnancy body mass index or family history of hypertension) received one additional star.

^c^ One star was awarded if there was no significant difference in the response rate between control subjects and cases in the chi-square test (P > 0.05).

^d^ The studies are considered to be low-quality, when the sores were lower than six stars in quality assessment.

**Supplementary Figure S1. Leave-one-out sensitivity analysis for the association between CDKAL1 rs7754840 polymorphism and GDM risk.**
